# Selected spices and their combination modulate hypercholesterolemia-induced oxidative stress in experimental rats

**DOI:** 10.1186/0717-6287-47-5

**Published:** 2014-03-26

**Authors:** Gloria A Otunola, Oyelola B Oloyede, Adenike T Oladiji, Anthony J Afolayan

**Affiliations:** Medicinal Plant and Economic Development Research Centre, Department of Botany, University of Fort Hare, Alice, 5700 South Africa; Department of Biochemistry, University of Ilorin, Ilorin, Nigeria; Department of Home Economics and Food Science, University of Ilorin, Ilorin, Nigeria

**Keywords:** Lipid peroxidation, Oxidative stress, Spices, Hypercholesterolemia, ROS

## Abstract

**Background:**

Effect of aqueous extracts of *Allium sativum* (garlic)*, Zingiber officinale* (ginger), *Capsicum fructensces* (cayenne pepper) and their mixture on oxidative stress in rats fed high Cholesterol/high fat diet was investigated. Rats were randomly distributed into six groups (n = 6) and given different dietary/spice treatments. Group 1 standard rat chow (control), group 2, hypercholesterolemic diet plus water, and groups 3, 4, 5, 6, hypercholesterolemic diet with 0.5 ml 200 mg · kg-1 aqueous extracts of garlic, ginger, cayenne pepper or their mixture respectively daily for 4 weeks.

**Results:**

Pronounced oxidative stress in the hypercholesterolemic rats evidenced by significant (p < 0.05) increase in MDA levels, and suppression of the antioxidant enzymes system in rat’s liver, kidney, heart and brain tissues was observed. Extracts of spices singly or combined administered at 200 mg.kg-1 body weight significantly (p < 0.05) reduced MDA levels and restored activities of antioxidant enzymes.

**Conclusions:**

It is concluded that consumption of garlic, ginger, pepper, or their mixture may help to modulate oxidative stress caused by hypercholesterolemia in rats.

## Backgroud

Hypercholesterolemia is a lipoprotein metabolic disorder characterized by high serum levels of low density lipoprotein (LDL-C) and blood cholesterol. Alteration in cholesterol and triglycerides metabolism as a result of hypercholesterolemia has been shown to negatively affect oxidative stress biomarkers and promotes production of reactive oxygen species (ROS) by various mechanisms, that leads to increased lipid peroxidation [1].

Oxidative stress is derivate from an imbalance between the generation of ROS and the endogenous antioxidant systems so that the latter is overwhelmed [2]. It has been reported to lead to increased lipid peroxidation, which in turn is involved in the aetiology of several chronic diseases [3]. The significant role of ROS in the pathogenesis of certain human diseases such as cardiovascular disease, cancer, arthritis and post-ischemic re-oxygenation injury is increasingly being recognized [4]. ROS are highly reactive molecules derived from normal essential metabolic processes in the human body. They could also come from external sources such as exposure to X-rays, ozone, cigarette smoking, alcohol and industrial chemicals [5].

According to several reports, controlling ROS is believed to be more effective in preventing cardiovascular diseases with more potential for success than restrictive fat and cholesterol diets [6–9]. The significant role of ROS in the induction of oxidative damage to biomolecules is increasingly being reported [4, 10]. Oxidative damage of cell membranes, DNA, and proteins induced by ROS has been implicated in the aetiology of several degenerative diseases like aging, cancer and cardiovascular diseases [11].

There is a growing interest in the mechanism of antioxidants in relation to damage caused by aggressive oxygen species and thiyl radicals. The main work of antioxidants seems to be scavenging these ROS and converting them to inactive substances [12]. Antioxidants are therefore crucial to the body’s multilevel defense against free radicals. Lines of defense include enzymes (glutathione reductase, glutathione peroxidase, catalase, superoxide dismutase), vitamins (C, E and carotenoids), polyphenolics and other bioactive compounds, [13–15].

Spices are reported to contain natural chemicals like vitamins, phytonutrients, mineral elements, alkaloids, flavonoids, terpenoids and sesquisterpenes, [16]. These chemicals confer on spices, excellent antioxidant properties that help to neutralize free radicals in the body and prevent lipid peroxidation and mutation of DNA and healthy cells into cancerous cells, [17].

Garlic (*Allium sativum*), ginger (*Zingiber officinale*) and cayenne pepper (*Capsicum fructensces*) are common culinary spices that have been reported to possess antioxidant properties. The prophylactic and therapeutic effects of dietary garlic, ginger and pepper in reducing the risks of cardiovascular diseases, atherosclerosis, bacterial infections, inflammation, as well as vasodilator, anti-diabetic, anti-ulcer and hypocholesterolemic agents amongst others have been reviewed [16, 18–21]. These spices are either used singly or combined in most cuisines all over the world. It is believed that when multiple antioxidants are used in combination, they prevent vulnerability to other agents and synergistically pool their antioxidant properties.

This study was therefore undertaken to assess the individual and combined additive or synergistic effects of the three spices- garlic, ginger and pepper on oxidative injury caused by dietary hypercholesterolemia in order to establish if combining the three will be more effective therapeutically using rat models.

## Results and discussion

**Figure 1:**
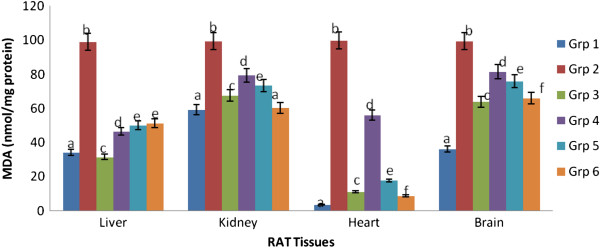
**Effect of extracts of spices on malondialdehyde content in hypercholesterolemia-induced oxidative stress in rat tissues.** ^a^Values are means ± SD (n=6). ^b^Bars with different superscripts within the same tissues are significantly different at p<0.05. ^c^Malondialdehyde is expressed per milligram of protein. ^d^Grp 1- control, Grp 2- hypercholesterolemic diet, Grp 3- hypercholesterolemic diet + garlic, Grp 4- hypercholesterolemic diet + ginger, Grp 5- hypercholesterolemic diet + pepper, Grp 6- hypercholesterolemic diet + mixture.

In this study, control rats fed normal diet were used as the reference point for each variable measured. Lipid peroxidation in the cardiac, hepatic, renal and brain tissues are presented in Figure 1. There was pronounced oxidative stress in the hypercholesterolemic rats as evidenced by significant (p < 0.05) increase in MDA levels in the liver, heart, kidney and brain tissues compared with the control group. MDA in all the tissues of hypercholesterolemic rats was significantly (p < 0.05) elevated compared to control. However, rats administered with 200 mg/kg body weight extract of any of the spices or their mixture exhibited significant (p < 0.05) decrease in MDA levels when compared with the hypercholesterolemic rats, with the mixture extract showing the best modulatory action.

**Figure 2:**
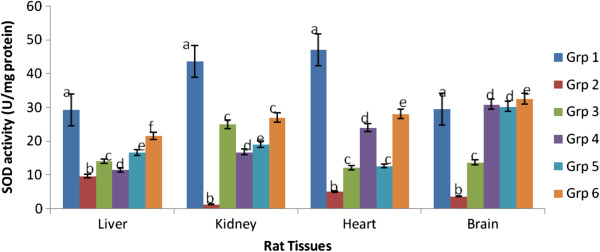
**Effect of extracts of spices on superoxide dismutase activity in hypercholesterolemia-induced oxidative stress in rat tissues.** ^a^Values are means ± SEM (n=6, p<0.05). ^b^Bars with the same colour but different letters are significantly different. ^c^Grp1-control, Grp 2- hypercholesterolemic diet, Grp 3- hypercholesterolemic diet + garlic, Grp 4- hypercholesterolemic diet + ginger, Grp 5- hypercholesterolemic diet + pepper, Grp 6- hypercholesterolemic diet + mixture of spices.

**Figure 3:**
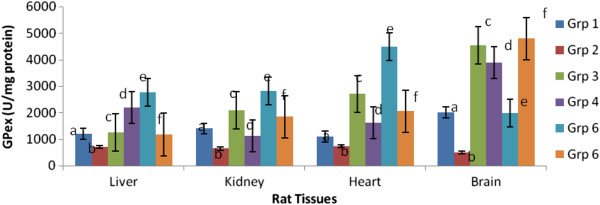
**Effect of extracts of spices on glutathione peroxidase activity in hypercholesterolemia-induced oxidative stress in rat tissues.** ^a^Values are means ±SEM (n=6) (p<0.05). ^b^Bars with the same colour but different letters are significantly different. ^c^Grp1- control, Grp 2- hypercholesterolemic diet, Grp 3- hypercholesterolemic diet + garlic, Grp 4- hypercholesterolemic diet + ginger, Grp 5- hypercholesterolemic diet + pepper, Grp 6- hypercholesterolemic diet + mixture of spices.

**Figure 4:**
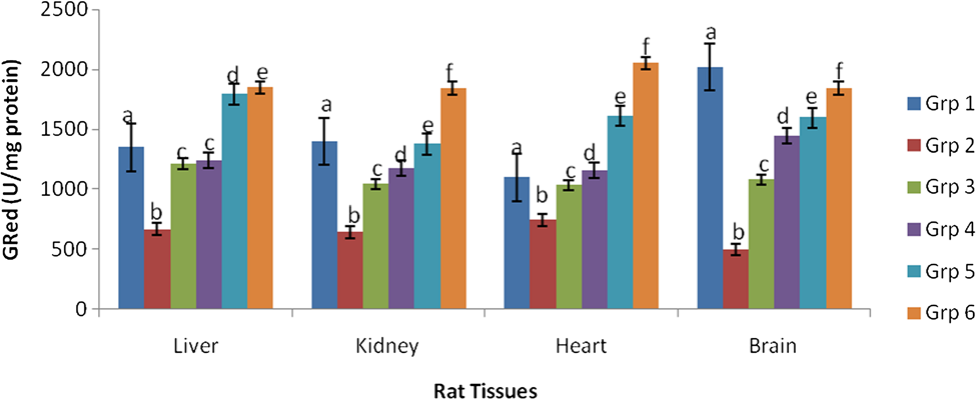
**Effect of extracts of spices on glutathione reductase activity in hypercholesterolemia-induced oxidative stress in rat tissues.** ^a^Values are means ± SEM (n=6), (p<0.05). ^b^Bars with the same colour but different letters are significantly different. ^c^Grp1-control, Grp 2-hypercholesterolemic diet, Grp 3-hypercholesterolemic diet + garlic, Grp 4- hypercholesterolemic diet + ginger, Grp 5-hypercholesterolemic diet + pepper, Grp 6- hypercholesterolemic diet + mixture of spices.

Figures 2, 3 and 4 depicts the altered activities of antioxidant enzymes namely SOD, GPex and GRed in the cardiac, hepatic, renal and brain tissues of rats. Compared to the control, the hypercholesterolemic rats showed a significant (p < 0.05) depression in SOD, GPex and GRed activities. Hypercholesterolemic rats treated with 200 mg.kg-1 body weight of the spice extracts exhibited a significant (p < 0.05) elevation of enzymes activities in all the tissues.

Hypercholesterolemic rats treated with extract of the mixture gave the best response for SOD and GRed indicating an additive effect of the three spices, while for GPex it was those treated with pepper extract that gave the best result.

Lipid peroxidation is a process associated with the pathogenesis of many chronic diseases such as atherosclerosis, cancer and diabetes. In this study, elevated cardiac, hepatic, renal and brain lipid peroxidation status in untreated hypercholesterolemic rats indicate increased oxidative injury and is consistent with reports from similar studies that showed that hypercholesterolemia leads to increased lipid peroxidation [22–24]. This effect was ameliorated following administration of 200 mg/kg body weight extract of the garlic, ginger, pepper and their mixture as evidenced by the reduced level of MDA. Ability of the spice extracts singly and combined to inhibit lipid peroxidation *in vivo* could be attributed to the free radical scavenging activities of their components especially phenols, flavonoids, and antilipidemic agents [25]. Similar observations were made by [26, 27] on the effect of cinnamon/garlic extracts and phenolic compounds from rosemary respectively on hypercholesterolemia induced oxidative stress in rats. Garlic, ginger and pepper have been reported to possess antioxidants especially flavonoids and phenolics which could account for their antioxidant activity [28–30].

Antioxidant enzymes such as SOD, GPex, and GRed catalyse the conversion of reactive oxygen species to harmless species. In this study, the reduced activities of hepatic, renal, cardiac and brain antioxidant enzymes in rats fed a hypercholesterolemic diet could be as a result of increased demand for these enzymes in deactivating the high influx of ROS generated by the high cholesterol diet, insufficiency of the enzymes, or failure of the antioxidant defence system to overcome the influx of reactive oxygen species [31, 32]. Administration of 200 mg/kg extracts of garlic, ginger, pepper or their mixture significantly improved the antioxidant enzymes activities by restoring them almost to normal levels in the rats treated. These findings agree with earlier reports by other investigators on modulation of hypercholesterolemia induced oxidative stress in rats using *Bacopa monniera,* ascorbic acid, rutin and mushrooms respectively [1, 31, 32].

## Conclusions

Findings of this study suggest that extracts of garlic, ginger and pepper singly or combined possess antioxidant activity. Administration of a 200 mg/kg body weight aqueous extract of any of the spices significantly reduced lipid peroxidation and modulated effect of hypercholesterolemia induced oxidative stress, as well as improved the antioxidant defence system.

The results here therefore suggest that dietary consumption of these spices may provide benefits for patients with hypercholesterolemia-induced oxidative stress. The health implications of these findings may however be limited by the small group of rats used and the fact that these results may not be extrapolated to human populations. More detailed clinical studies involving human models may therefore be necessary.

## Methods

### Preparation of spice extracts

Garlic *(Allium sativum*), ginger (*Zingiber officinale*) and pepper (*Capsicum fructensces*) obtained from the local market in Ilorin, Nigeria, were individually sorted to remove grits and dirt, washed, thinly sliced, then oven-dried at 60°C for 72 h. The dried spices were milled into fine powder, packed into airtight plastic bottles and stored in a cold room at -20°C until needed. From the powdered samples, 50 g of each spice was extracted with 1 L of distilled water and boiled for 10 min at 100°C. It was allowed to cool, filtered and freeze-dried (Vir Tis bench top K, Vir Tis, Gardiner, New York, USA) for 48 h. To prepare a mixture of the spices, equal amounts of each spice (50:50:50 g), were weighed and thoroughly mixed together by passing through a coffee grinder of a home blender set. From the mixture, 50 g was taken and extracted in like manner as described above. The freeze-dried samples were stored in airtight plastic bottles at 4°C.

Dose equivalent to 200 mg of each spice and their combination extract per kg body weight of rat was calculated and reconstituted in distilled water. These dose is consistent with reports from previous studies [33–36].

### Housing and management of animals

Thirty six (36) male rats of Wistar strain weighing 168 ± 10 g were randomly distributed into six groups, with six animals (n = 6) in each group after one week of acclimatization. They were exposed to a 12 h light/dark cycle, constant temperature (22°C ± 2°C) and humidity (70% ± 4%) with water and feed made available *ad libitum*. The animals were handled according to the guidelines of the National Research Council Guide for the Care and Use of Laboratory Animals [37] and the study was approved by the Animal Ethics Committee, University of Fort Hare, Alice, South Africa.

### Experimental protocols

Two diets, namely standard rat chow and hypercholesterolemic diet (1% cholesterol and 25% soybean oil) that has been established to induce hypercholesterolemia [38, 39] were used. The diets were kept refrigerated and used within 1 week of preparation. Rats in group 1 (positive control), were fed the standard rat chow with water *ad libitum*. Group 2 (negative control) were fed the hypercholesterolemic diet and water, while groups 3, 4, 5, and 6 rats were fed the hypercholesterolemic diet and administered daily with aqueous extracts of garlic, ginger, pepper or their mixture at a dose equivalent to 200 mg/kg body weight of rats respectively.

At the end of 4 weeks, all the animals were fasted overnight and anaesthetized by intraperitoneal injection of pentobarbital sodium (45 mg/kg). Blood samples were collected by cardiac puncture into vacutainers (CE Sterile R 456018P, greiner.bio.one, United Kingdom) for various biochemical analyses. The rats were finally euthanized and the heart, liver and kidney excised and rinsed in ice-cold saline to maintain physiologic status.

### Determination of lipid peroxidation and antioxidant enzymes activities in liver, kidney, heart and brain of rats

Lipid peroxidation in the organs was determined by estimating levels of malondialdehyde (MDA) as described by [40]. This method was used to measure spectrophotometrically the color produced by the reaction of TBA with malondialdehyde at 532 nm using a Randox chemical assay kit. Tissue supernatants (50 μl) were added to test tubes containing 2 μl of butylated hydroxytoluene (BHT) in methanol. Next, 50 μl of acid reagent (1 M phosphoric acid) was added and finally 50 μl of TBA solution was added. The tubes were mixed vigorously and incubated for 60 min at 60°C. The mixture was centrifuged at 10,000 × g for 3 min. The supernatant was put into microplate wells in aliquots of 75 μl, and absorbance was measured with a microplate reader at 532 nm. TBARS levels were expressed as nmol/mg protein in various organs (brain, liver, heart and kidney).

Activities of superoxide dismutase (SOD), glutathione peroxidase (GPex) and glutathione reductase (GRed) were assayed in the liver, heart, kidney and brain of experimental rats.

SOD accelerates the dismutation of the toxic superoxide radical (O_2_^.^) produced during oxidative energy processes to hydrogen peroxide and molecular oxygen. SOD was determined using Randox kits as described by [41]. The method uses xanthine and xanthine oxidase to generate superoxide radicals which reacts with 2-(4-iodophenyl)-3-(4-nitrophenol)-5-phenyltetrazolium chloride (I.N.T) to form a red formazan dye which absorbs at 505 nm. A 30 μl of sample was pipetted into a cuvette with 1000 μl of buffer (reagent 1) R1, and mixed well. To this mixture, 150 μl of xanthine oxidase, (reagent 2) R2 was added and mixed, absorbance was read at 505 nm.

Gpex activity was determined using Randox assay kit as described by the manufacturer based on the method of [42]. Glutathione peroxidase catalyses oxidation of glutathione by cumene hydroperoxide. In the presence of glutathione reductase and NADPH, the oxidized glutathione (GSSG) is immediately converted to the reduced form with a concomitant oxidation of NADPH to NADP^+^. An aliquot (10 μl) of the sample was added to 500 μl of reagent 1 (R_1_) and mixed. To this mixture was added 20 μl of cumene (R_2_ reagent 2). A blank was set up using 10 μl of distilled water and absorbance was read at 340 nm.

GRed activity was determined as described by [43] using Randox kits. Glutathione reductase catalyses the reduction of glutathione (GSSG) in the presence of NADPH, which is oxidized to NADP^+^. The decrease in absorbance of the sample is measured at 340 nm. 10 μl of tissue sample was mixed with 250 μl of substrate R_1_ (reagent 1) after which 50 μl of R_2_ (reagent 2) was added, mixed and the timer started. Initial absorbance was read at1 min, read again after 2, 3, 4, and 5 min.

### Statistical analysis

Data were expressed as means ± SEM from 6 determinations. Both descriptive and inferential statistical methods were used for data analysis. When the data were subjected to prior investigations before analysis, parametric assumptions including homoscedasticity, normality and independence of observations were satisfied. Hence, a parametric test (analysis of variance, ANOVA) was used to test for statistical significance among the treatment means. Differences among treatment means were considered significant at P < 0.05. Means that were significantly different from each other were segregated using Duncan’s Multiple Range Test. All analyses were done using MINITAB (Student Version 12 for Windows) Software.
